# Quantitative Analysis of Matrine in Liquid Crystalline Nanoparticles by HPLC

**DOI:** 10.1155/2014/368682

**Published:** 2014-04-14

**Authors:** Xinsheng Peng, Baohong Li, Min Hu, Yahao Ling, Yuan Tian, Yanxing Zhou, Yanfang Zhou

**Affiliations:** Guangdong Medical College, Xincheng Avenue, Guangdong 523808, China

## Abstract

A reversed-phase high-performance liquid chromatographic method has been developed to quantitatively determine matrine in liquid crystal nanoparticles. The chromatographic method is carried out using an isocratic system. The mobile phase was composed of methanol-PBS(pH6.8)-triethylamine (50 : 50 : 0.1%) with a flow rate of 1 mL/min with SPD-20A UV/vis detector and the detection wavelength was at 220 nm. The linearity of matrine is in the range of 1.6 to 200.0 **μ**g/mL. The regression equation is *y* = 10706*x* − 2959 (*R* ^2^ = 1.0). The average recovery is 101.7%; RSD = 2.22%  (*n* = 9). This method provides a simple and accurate strategy to determine matrine in liquid crystalline nanoparticle.

## 1. Introduction

Matrine is an alkaloid isolated from* sophora flavescens* ait. It exhibits broad bioactivity including inducing apoptosis of scar cells [1] and anti-inflammatory [2] and anticancer [3, 4] properties, which have been used in the traditional Chinese medicine for treatment of hypertrophic scar, inflammation, cancer, and other diseases without obvious toxicity or side effect in clinic. Several preparions, hydrogel [5], lotion [6], injection [7], and liposome [8], for example, have been reported in the literature. We are further interested in the form of liquid crystalline nanoparticles (cubosome), for cubosome consists of a curved bicontinuous lipid bilayer extending in three dimensions and separating two congruent networks of water channels [9–11], which can enclose hydrophilic, amphiphilic, and hydrophobic substances ranging from low-molecular-weight drugs to proteins, peptides, amino acids, and nucleic acids [12]. Compared to liposomes, cubosomes showed better storing stability at room temperature and could endure heat treatment [13–15]. Cubosomes could exist at almost any dilution level in water and drug leakage was less concerned compared with liposome. Gan et al. [16] reported that cubosomes had a higher permeability coefficient (4.5-fold) compared to eye drops when dexamethasone was used as a model drug. All these properties are favorable for drug delivery. Therefore, we consider that the cubosome might represent a promising vehicle containing matrine for effective ocular drug delivery.

HPLC, LC/MS/MS, and ESI-QTOF-MS/MS methods have been used to determine matrine in samples at present [6, 17, 18]. However, no detection methods have been developed to determine matrine in cubosomes. The aim of this study is to establish and validate a simple, sensitive, and accurate HPLC method to determine matrine combined in liquid crystalline nanoparticles.

## 2. Experiment

### 2.1. Reagents and Chemicals

Glycerol monooleate (DIMODAN MO/D KOSHER, material number 116703) was kindly provided by Danisco Cultor (Brabrand, Denmark) and used as received. Poloxamer 407 (PEO98POP67PEO98) was a gift from BASF (Ludwigshafen, Germany). Matrine was purchased from Shenzhen Yihao Technology Development Co., Ltd. (Guangdong, China) with purity over 98.0%. Methanol (chromatographic grade) was purchased from Tianjin Fuyu Chemical Co., Ltd. (Tianjin, China). Milli-Q-grade water purified through a Millipore system (ELGA LabWater, Sartorius, UK) was used throughout this study. PBS (pH 6.8) was made according to the Chinese Pharmacopoeia (2010). All other reagents were of analytical grade and used as received.

### 2.2. Preparation of Matrine Liquid Crystal Nanoparticles [19, 20]

Liquid crystal nanoparticles were prepared through the fragmentation of glycerol monooleate/poloxamer 407 bulk cubic gels. Glycerol monooleate (3 g) and poloxamer 407 (300 mg) were first melted at 60°C in a hot water bath until they were homogeneous, after which matrine was added to dissolve/blend under continuous stirring. Water (6.7 mL) was then added gradually and the mixture was vortex-mixed to achieve a homogeneous state. After equilibration for 48 hours at room temperature, the cubic phase gel was formed. By adding 20 mL of water, the cubic gel was disrupted by mechanical stirring. Subsequently, the crude dispersion was fragmented for 10 min by intermittent probe sonication (JY-96 IIN, Ningbo Scientz Biotechology Co., Ltd, China) at 200 W energy input using a pulse mode (9-second pulses interrupted by 18-second breaks) under cooling in a 20°C water bath. The resulting milky coarse dispersion was homogenized using a high-pressure homogenizer (Avestin Em-C3, Ottawa, Canada) at certain high pressures and cycles to obtain an opalescent dispersion of the cubic nanoparticles. The final dispersion of liquid crystal nanoparticles was stored at room temperature for further studies.

### 2.3. Liquid Chromatographic Conditions

The HPLC analysis was carried out using a Shimadzu system that is equipped with an LC-20AT pump, SPD-20A UV/vis detector connected to Shimadzu Spin Chrome software. The chromatographic assay was performed on a reversed-phase ODS-BP C18 column (5 *μ*m, 4.6 mm × 250 mm) at ambient temperature 25°C. An injection volume of 10 *μ*L was injected into the HPLC system. The mobile phase under isocratic mode was a mixture of methanol-PBS (pH 6.8)-triethylamine (50 : 50 : 0.1%, v/v). The mobile phase was degassed by an ultrasonic bath and filtered with 0.45 *μ*m membrane under vacuum. The flow rate was 1.0 mL/min. Quantification of matrine was performed at 220 nm. The chromatographic run time was 20 min. All the calculations concerning the quantitative analysis were carried out by an external standard method based on peak areas.

### 2.4. Preparation of Standard Stock Solutions and Working Solutions

To prepare the stock solution, matrine (10 mg) was accurately weighed into 50 mL volumetric flask, made up to volume with methanol, and then the volume was adjusted to 50 mL. This solution was further diluted with methanol to yield solutions containing 100.0, 50.0, 25.0, 12.5, 6.3, 3.1, and 1.6 *μ*g/mL. The chromatogram peak area of each known concentration was calculated. Results from each analysis were subjected to regression analysis.

### 2.5. Quantification of Matrine in Liquid Crystalline Nanoparticles

0.2 mL (or 200.0 mg) of the nanoparticles was accurately transferred into a 10 mL volumetric flask, dissolved, and made up to volume with methanol. Then, the sample solutions were filtered using a 0.45 *μ*m filter membrane and injected (10 *μ*L) into the HPLC system three times under optimized chromatographic conditions. Matrine concentrations of the samples were determined by interpolation from calibration plots previously obtained.

### 2.6. Method Validation

The method was validated in terms of parameters of specificity, linearity, sensitivity, accuracy, precision, and reproducibility according to the International Conference on Harmonisation.

The specificity of the method was assessed by comparing chromatograms of matrine working solution, blank excipients sample without matrine, and equal concentrations samples of compound liquid crystalline nanoparticles made as the previous procedure. All the samples were analyzed and recorded to ensure the absence of interfering peaks.

The linearity of the method was studied by injecting seven known concentrations of the standard in the range of 1.6–200 *μ*g/mL. The responses were measured as peak area.

The sensitivity of the method was evaluated with limit of detection (LOD) and limit of quantification (LOQ). LOD and LOQ were established at a signal-to-noise ratio (S/N) of 3 and 10, respectively.

The accuracy of the method was tested by comparing the percent analyte recovered by the optimum method at three concentration levels (80.0, 100, and 120.0 *μ*g/mL).

Precision was demonstrated at 3 concentration levels in intraday and interday studies. Intraday precision was determined by injection of standard solutions of matrine at 3 concentration levels (50, 25, and 12.5 *μ*g/mL), on the same day. Interday precision was checked by repeating the studies on two different days.

## 3. Results

### 3.1. Method Validation

**Figure 1:**
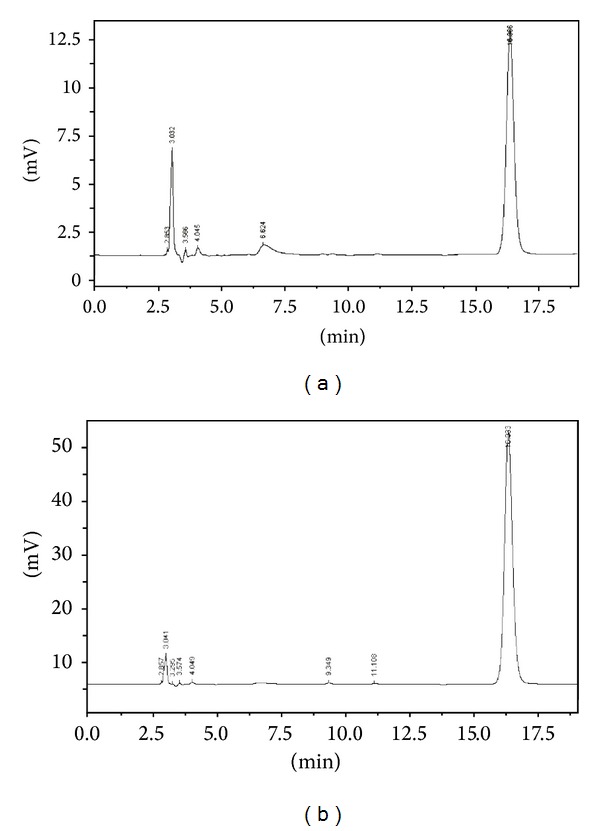
HPLC chromatogram of standard matrine (a) and sample (b).

The specificity was evaluated by analyzing blank excipients sample, matrine standard solution, and liquid crystalline nanoparticles samples. The typical HPLC chromatograms under optimum conditions were shown in Figure 1. The retention times of matrine at a flow rate of 1.0 mL/min was 16.3 min. Analyte peaks were well resolved and free from tailing (<1.5). The excipients did not interfere with the detection of matrine.

The calibration curves for matrine were found to be linear within the range of 1.6 to 200.0 *μ*g/mL. The regression equation was *y* = 10706*x* − 2959 (*R* ^2^ = 1.0), where *y* is peak area and *x* is the concentration (*μ*g/mL) of matrine standard solution. The correlation coefficient indicated a good linear relationship between peak area and concentration over a wide range.

The LOD (signal/noise ratio of 3 : 1) was calculated as 1.3 × 10^−1^ *μ*g/mL and the LOQ (signal/noise ratio of 10 : 1) was determined as 3.9 × 10^−1^ *μ*g/mL.

Mean recovery for matrine at three concentration levels (80.0, 100, and 120.0 *μ*g/mL) was found to be 102.1 ± 1.9% (RSD = 1.96%, *n* = 3), 102.6 ± 1.9% (RSD = 2.94%, *n* = 3), 100.5 ± 2.1% (RSD = 2.12%, *n* = 3), respectively. The intra- and interday RSD values were lower than 1.0%. The low values of RSD revealed satisfactory precision and accuracy of this present method.

### 3.2. Content of Matrine in Liquid Crystalline Nanoparticles

Matrine was determined with the proposed method in liquid crystalline nanoparticles. The mean concentration was 9.5 mg/mL (RSD = 1.4%, *n* = 3).

## 4. Discussion

**Figure 2:**
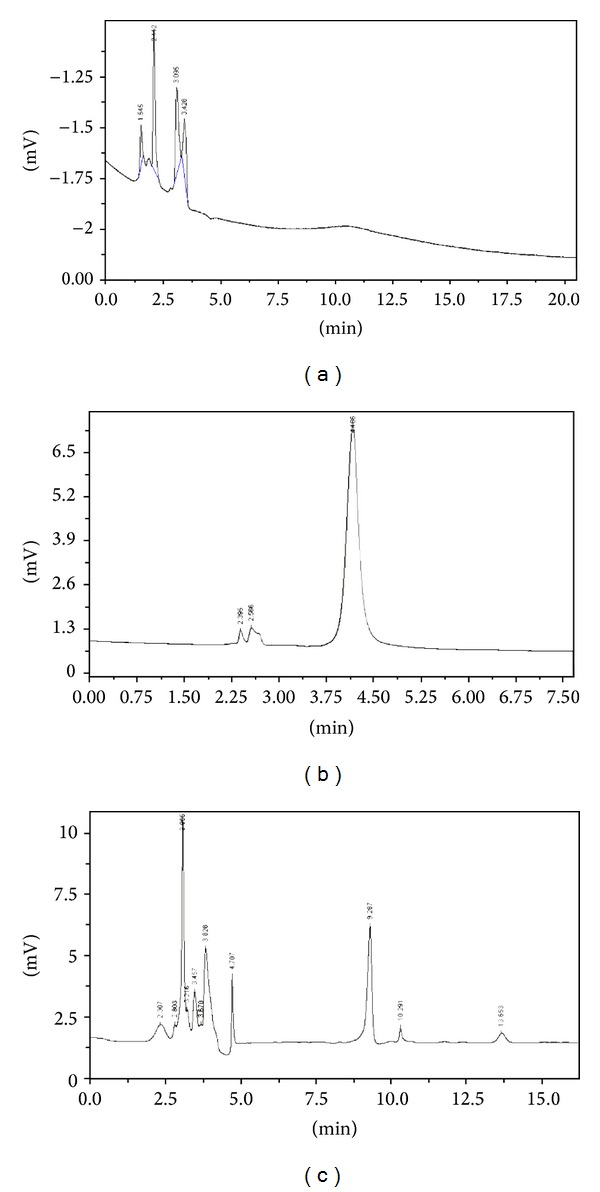
HPLC chromatogram of standard matrine in different mobile phase condition. (a) Methanol: water = 14 : 86; (b) methanol: 0.01 mol/LKH_2_PO_4_ = 20 : 80 (PH = 6~7); (c) acetonitrile: anhydrous ethanol: 3% H_3_PO_4_ = 80 : 10 : 10.

Several mobile phase systems including methanol-water, ethanol-wate-KH_2_PO_4_, and acetonitrile-ethanol-H_3_PO_4_ systems have been tested in this study. However, the chromatogram of standard matrine might disappear or appear in a wide range with inaccurate calculation area (see Figure 2). There were more than three peaks in acetonitrile-ethanol-H_3_PO_4_ system. Matrine crystalline has 4 forms, namely, *α*, *β*, *γ*, and *δ* matrine. The incorrect mobile phase may change the matrine solution to nanocrystalline because of the solubility.

The chromatographic method was eventually carried out using an isocratic system with a mobile phase of methanol-PBS (pH 6.8)-triethylamine (50 : 50 : 0.1%) applied at a flow rate of 1 mL/min with detection wavelength at 220 nm. Under these optimum mobile phase conditions, elution of analyte was completed in less than 20.0 min and retention time of matrine was 16.3 min. The method was validated according to ICH guidelines with the parameters of specificity, linearity, sensitivity, accuracy, precision, and reproducibility. All data shows the method is accurate within the desired ranges.

## 5. Conclusion

A simple, rapid, selective, and sensitive HPLC method has been developed and validated for the determination of matrine when formulated in cubosome particles. The present study is the first report on the matrine determination combined with particle dispersion system. The method can be used for controlling the quality of the cubosome and helpful for further investigation.
